# Retrospective analysis of antitumor effects and biomarkers for nivolumab in NSCLC patients with *EGFR* mutations

**DOI:** 10.1371/journal.pone.0215292

**Published:** 2019-04-12

**Authors:** Miyuki Sato, Satoshi Watanabe, Hiroshi Tanaka, Koichiro Nozaki, Masashi Arita, Miho Takahashi, Satoshi Shoji, Kosuke Ichikawa, Rie Kondo, Nobumasa Aoki, Masachika Hayashi, Yasuyoshi Ohshima, Toshiyuki Koya, Riuko Ohashi, Yoichi Ajioka, Toshiaki Kikuchi

**Affiliations:** 1 Department of Respiratory Medicine and Infectious Diseases, Niigata University Graduate School of Medical and Dental Sciences, Niigata City, Niigata, Japan; 2 Department of Internal Medicine, Niigata Cancer Center Hospital, Niigata City, Niigata, Japan; 3 Histopathology Core Facility, Niigata University Faculty of Medicine, Niigata City, Niigata, Japan; 4 Division of Molecular and Diagnostic Pathology, Niigata University Graduate School of Medical and Dental Sciences, Niigata City, Niigata, Japan; National Cancer Center, JAPAN

## Abstract

Although the blockade of programmed cell death 1 (PD-1)/PD-ligand (L) 1 has demonstrated promising and durable clinical responses for non-small-cell lung cancers (NSCLCs), NSCLC patients with epidermal growth factor receptor (*EGFR*) mutations responded poorly to PD-1/PD-L1 inhibitors. Previous studies have identified several predictive biomarkers, including the expression of PD-L1 on tumor cells, for PD-1/PD-L1 blockade therapies in NSCLC patients; however, the usefulness of these biomarkers in NSCLCs with *EGFR* mutations has not been elucidated. The present study was conducted to evaluate the predictive biomarkers for PD-1/PD-L1 inhibitors in *EGFR*-mutated NSCLCs. We retrospectively analyzed 9 patients treated with nivolumab for *EGFR*-mutated NSCLCs. All but one patient received EGFR-tyrosine kinase inhibitors before nivolumab treatment. The overall response rate and median progression-free survival were 11% and 33 days (95% confidence interval (CI); 7 to 51), respectively. Univariate analysis revealed that patients with a good performance status (*P* = 0.11; hazard ratio (HR) 0.183, 95% CI 0.0217 to 1.549), a high density of CD4^+^ T cells (*P* = 0.136; HR 0.313, 95% CI 0.045 to 1.417) and a high density of Foxp3^+^ cells (*P* = 0.09; HR 0.264, 95% CI 0.0372 to 1.222) in the tumor microenvironment tended to have longer progression-free survival with nivolumab. Multivariate analysis revealed that a high density of CD4^+^ T cells (*P* = 0.005; HR<0.001, 95% CI <0.001 to 0.28) and a high density of Foxp3^+^ cells (*P* = 0.003; HR<0.001, 95% CI NA) in tumor tissues were strongly correlated with better progression-free survival. In contrast to previous studies in wild type *EGFR* NSCLCs, PD-L1 expression was not associated with the clinical benefit of anti-PD-1 treatment in *EGFR*-mutated NSCLCs. The current study indicated that immune status in the tumor microenvironment may be important for the effectiveness of nivolumab in NSCLC patients with *EGFR* mutations.

## Introduction

Lung cancer is the most common cause of cancer death worldwide [1, 2], and non-small-cell lung cancer (NSCLC) accounts for the most cases. Immunotherapy for NSCLCs has recently evolved into a new stage of a novel modality with immune-checkpoint inhibitors (ICIs) [3]. For example, anti-programmed-cell death-1 (PD-1) and anti-PD-ligand (L) 1 antibodies have demonstrated promising and durable responses across a broad range of solid tumors, including NSCLCs [4].

Recent studies have reported the possible predictive biomarkers for PD-1/PD-L1 blockade therapies. The expression of PD-L1 on tumor cells is the most commonly examined biomarker. Subgroup analyses in a large phase III study investigating nivolumab in nonsquamous lung cancer showed a correlation between overall survival (OS) and PD-L1 expression on tumor cells [5]. Compared to platinum-doublet chemotherapy, pembrolizumab significantly prolonged progression-free survival (PFS) and OS in NSCLC patients with a high expression of PD-L1 [6]. Other predictive biomarkers, such as tumor-mutation burden, tumor-infiltrating lymphocytes (TILs) including CD8^+^ T cells and regulatory T cells (Tregs), neutrophil-to-lymphocyte ratio (NLR) in peripheral blood, and frequency of immune-suppressive cells in peripheral blood and tumor tissues have been evaluated to select patients who are more likely to respond to ICIs [7–12].

Excellent therapeutic effects of epidermal growth factor receptor-tyrosine kinase inhibitors (EGFR-TKIs) have been reported in *EGFR* mutation-positive NSCLCs [13–20]. However, EGFR-TKIs do not cure NSCLCs. All treated patients eventually develop resistance to EGFR-TKIs, and the illness advances. New therapeutic strategies need to be established for *EGFR*-mutated patients. In therapy with ICIs, a clinical study showed no survival benefit of nivolumab in patients with *EGFR* mutations [5]. Similarly, compared with docetaxel, pembrolizumab did not show any survival advantage in *EGFR*-mutated NSCLCs [21]. NSCLCs harboring *EGFR* mutations are associated with the low effectiveness of treatments with PD-1/PD-L1 inhibitors [22, 23]. Possible mechanisms could be the poor antigenicity of tumors due to a low tumor mutation burden and the immunosuppressive microenvironment in tumor tissues; however, the reasons why PD-1/PD-L1 blockade therapies failed to show a survival benefit in *EGFR*-mutated NSCLCs are not fully understood [8, 24, 25]. Furthermore, the effectiveness of PD-1/PD-L1 blockade therapies in *EGFR*-mutated NSCLC patients with predictive biomarkers for ICIs remains to be elucidated.

This study aimed to evaluate the potential predictive biomarkers for nivolumab in NSCLC patients with *EGFR* mutations.

## Materials and methods

### Patients

We retrospectively analyzed the data of consecutive patients who received nivolumab for advanced NSCLC in the Niigata Cancer Center Hospital and Niigata University Medical and Dental Hospital between January 2016 and December 2017. *EGFR* mutation testing was performed using the peptide nucleic acid–locked nucleic acid polymerase chain reaction clamp method or the PCR-invader method [26, 27]. Patients received nivolumab (3 mg/kg) intravenously every 2 weeks until disease progression or unacceptable toxic effect. The present study was conducted in accordance with the Helsinki Declaration of the World Medical Association. The protocol was approved by the institutional review board of the Niigata University Medical and Dental Hospital and the Niigata Cancer Center Hospital and written informed consent was waived because of the retrospective design.

### Immunohistochemistry

In this study, tumor tissues that were adequate for immunohistochemistry analyses were required for all patients. Formalin-fixed, paraffin embedded tissue (FFPE) sections of 4-μm thickness were stained for PD-L1 using an automated immunohistochemistry assay (PD-L1 IHC 28–8 pharmDx, Agilent Technologies, Santa Clara, CA). PD-L1 expression on the tumor cell membrane was evaluated in sections including at least 100 tumor cells. To evaluate the expression of CD3, CD4, CD8 and Foxp3 in tumor-infiltrating lymphocytes, FFPE sections were deparaffinized and heated in an antigen retrieval solution at pH 9.0 (Nichirei Biosciences, Inc., Tokyo, Japan) for 15 min at 121°C. Endogenous peroxidase activity was quenched using 3% H_2_O_2_-methanol for 15 min, and then the sections were blocked with 10% normal goat serum. Next, sections were incubated with the primary antibodies for CD3 (clone PS1, Nichirei Corporation Tokyo, Japan), CD4 (clone 4B12, Nichirei Corporation, Tokyo, Japan), CD8 (clone C8/144B, Nichirei Corporation, Tokyo, Japan) and Foxp3 (clone 236A/E7, Abcam, Cambridge, UK) overnight incubation at 4°C. As the second step, a Histofine Simple Stain MAX-PO (multi) kit (Nichirei Corporation, Tokyo, Japan) was reacted for 30 min. The samples were carefully washed three times with phosphate-buffered saline (pH 7.4) in each step. To visualize antigen-antibody complex, a Histofine DAB substrate kit (Nichirei Corporation, Tokyo, Japan) was used. Nuclear staining was performed with hematoxylin. The numbers of CD4-, CD8-, Foxp3- and CD3-positive T cells were counted at 1 mm^2^ magnification in three different regions of the tumor and averaged, and the standard deviation calculated. The cell count was performed by using ImageJ software (National Institutes of Health) [28].

### Statistical analysis

Kaplan-Meier survival curves were constructed for PFS and OS, and differences between groups were identified using the log-rank test. Analysis was two-sided, with a 5% significance level and a 95% confidence interval (CI). All statistical analyses were performed using JMP 9.0.2 statistical software (SAS Institute, Cary, NC, USA).

## Results

### Patients’ characteristics

We retrospectively identified 9 patients with *EGFR-*mutated NSCLCs treated with nivolumab between March 2016 and September 2017. The patient characteristics are listed in Table 1. There were 6 females and the median age of all the patients was 62 years old (range, 37–72 years). Seven and 2 patients had a performance status of 1 and 2, respectively, and all patients had adenocarcinoma in histology. Five patients had an exon 19 deletion, one had an exon 19 deletion with T790M, one had L858R with T790M, one had an exon 20 insertion, and one had a S768I point mutation.

**Table 1:** Base line characteristics of all study patients (n = 9).

Parameter	Number of patients	%
Gender		
Female	6	67
Male	3	33
Median age (range), years	62	(37–72)
ECOG PS at initiation of nivolumab		
0 / 1 / 2	0 / 7 / 2	0 / 78 / 22
Smoking status		
Never smoked	7	78
Current or former	2	22
Histology		
Adenocarcinoma	9	100
Clinical stage		
IV	7	78
Post operative	2	22
Type of *EGFR* mutation		
Exon19 deletion	5	56
Exon19 deletion + T790M	1	11
L858R + T790M	1	11
Exon 20 insertion	1	11
S768I	1	11
Biopsy site		
Primary lesions	7	78
Lymph nodes	2	22
No. of prior regimens before nivolumab		
1 / 2 / ≥3	2 / 4 / 3	22 / 44 / 33

PS, performance status; EGFR, epidermal growth factor receptor.

### Clinical efficacy of nivolumab in *EGFR*-mutated NSCLC patients

During treatment with nivolumab, one patient achieved a partial response; however, 7 patients had progressive disease. The patient who responded to nivolumab had an exon 20 insertion and had not received EGFR-TKI treatment before nivolumab. We could not evaluate antitumor effects in one patient because the patient discontinued nivolumab treatment due to ileus and received osimertinib immediately (Table 2).

**Table 2:** Summary of responses.

	N = 9	%
CR	0	0
PR	1	11
SD	0	0
PD	7	78
NE	1	11
ORR		11
DCR		11
Cycles received median (range)	3	(2–41)

CR, complete response; PR, partial response; SD, stable disease; PD, progressive disease; NE, not evaluable; ORR, overall response rate; DCR, disease control ratio

**Fig 1:**
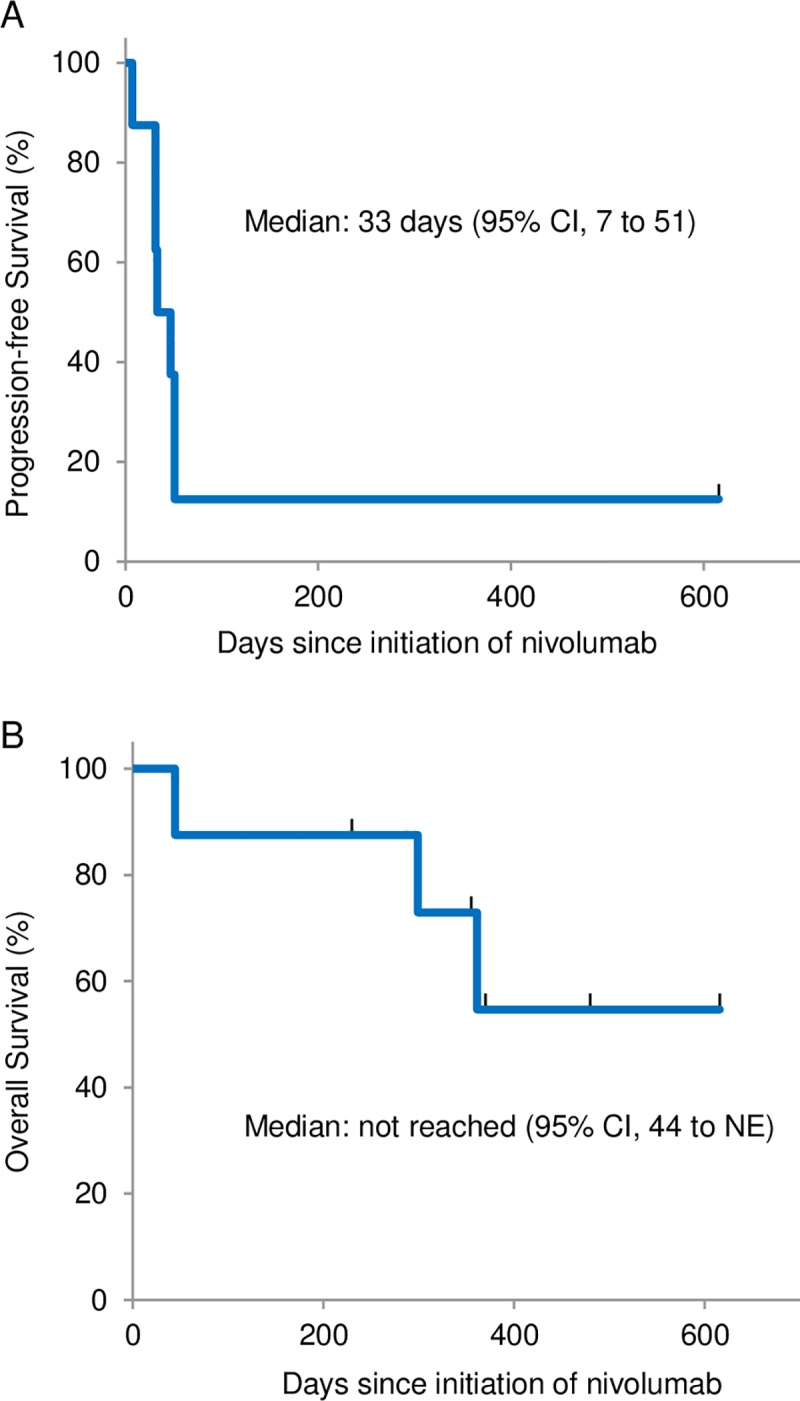
Survival curves of *EGFR*-mutated NSCLC patients treated with nivolumab. (A) Progression-free survival and (B) overall survival of patients treated with nivolumab.

The median number of treatment cycles was 3. The median PFS from the beginning of nivolumab was 33 days (95% CI 7 to 51), and the median OS was not reached (95% CI 44 to N.E.) (Fig 1).

### Biomarkers for nivolumab in *EGFR*-mutated patients

**Fig 2:**
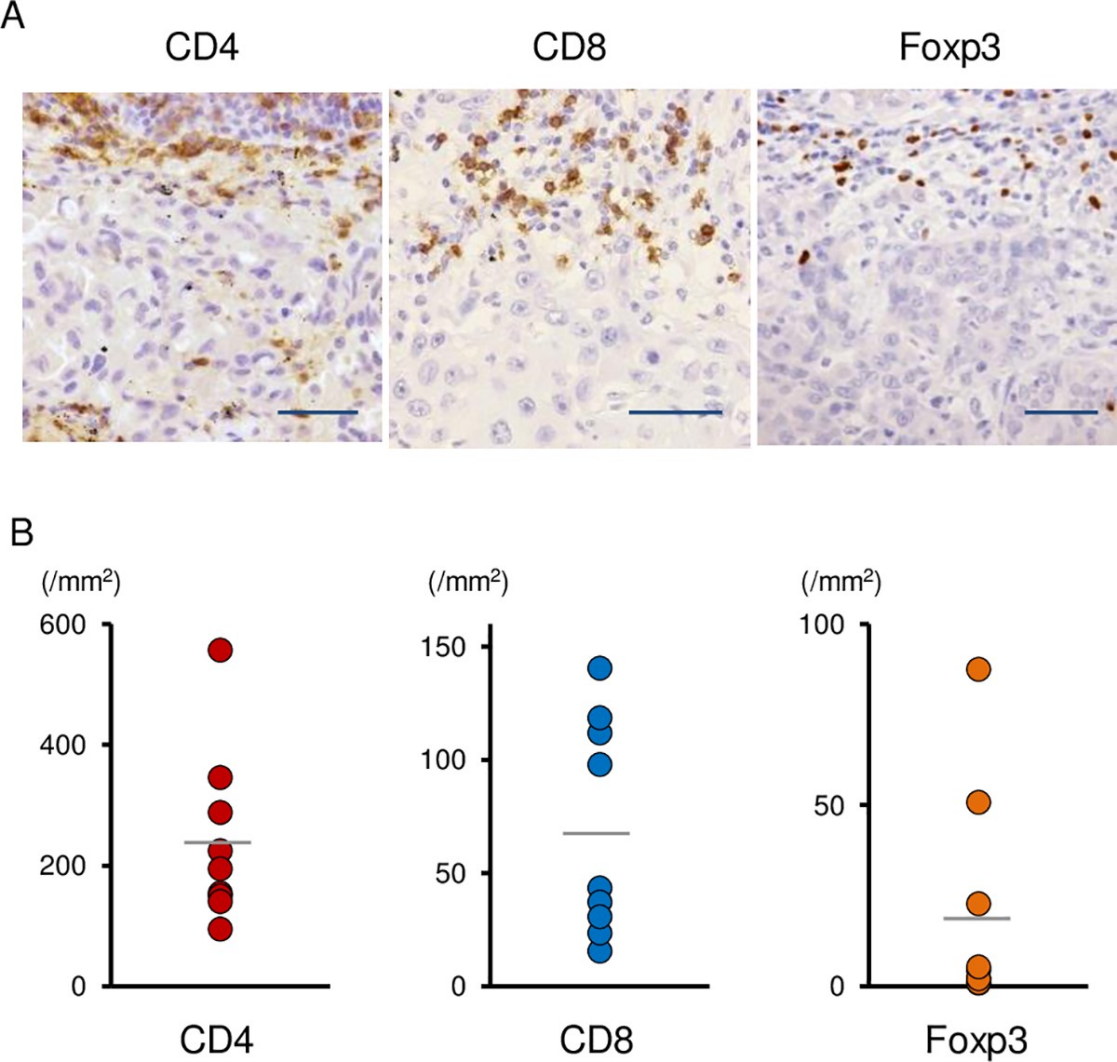
Immunohistochemical staining for CD4^+^, CD8^+^ and Foxp3^+^ TILs. (A) Representative examples of immunohistochemical staining images (scale bar 100 μm) for CD4, CD8 and Foxp3 are shown. (B) The numbers of CD4^+^ TILs, CD8^+^ TILs and Foxp3^+^ TILs are shown.

Next, we investigated whether the existence of potential biomarkers for immune-checkpoint inhibitors were associated with the therapeutic effects of nivolumab in NSCLC patients with *EGFR* mutations. In the current study, tumor tissues were obtained from all 9 patients and stained for IHC. Seven out of 9 patients received EGFR-TKIs before nivolumab and tumor tissues were obtained from these 7 patients after failure of EGFR-TKI treatment. Representative results of IHC are shown in Fig 2. Univariate analysis revealed that patients with good performance status (PS) and high numbers of CD4^+^ TILs (mean ≥ 239 /mm^2^) and Foxp3^+^ TILs (mean ≥ 20 /mm^2^) were likely to have a better PFS (Table 3). When these variables were included in the Cox proportional hazards model, a high number of CD4^+^ TILs and Foxp3^+^ TILs had significant hazard ratios for PFS (Table 3).

**Table 3:** Univariate and multivariate analyses by the Cox proportional hazards model (n = 9).

		Univariate			Multivariate		
		HR	95% CI	P	HR	95% CI	P
PD-L1	(≥1/<1)	0.801	0.16–3.384	0.766	0.397	0.013–5.039	0.481
CD4	(High/low)	0.313	0.045–1.417	0.136	<0.001	<0.001–0.28	0.005
CD8	(High/low)	0.552	0.11–2.326	0.419	4.732	0.415–157.678	0.219
Foxp3	(High/low)	0.264	0.0372–1.222	0.09	<0.001	NA	0.003
PS	(0, 1/≥2)	0.183	0.0217–1.549	0.11	1.009	0.096–11.386	0.994
NLR	(High/low)	7.462	0.951–151.145	0.559			
Smoking status	(Former/never)	2.305	0.315–12.351	0.366			
Age	(≥70/<70)	0.966	0.152–18.684	0.975			

HR, hazard ratio; C.I., Confidence interval; PD-L1, programmed cell death ligand 1; Treg, regulatory T cell; NA, not applicable; PS, performance status; NLR, neutrophil-to-lymphocyte ratio

**Fig 3:**
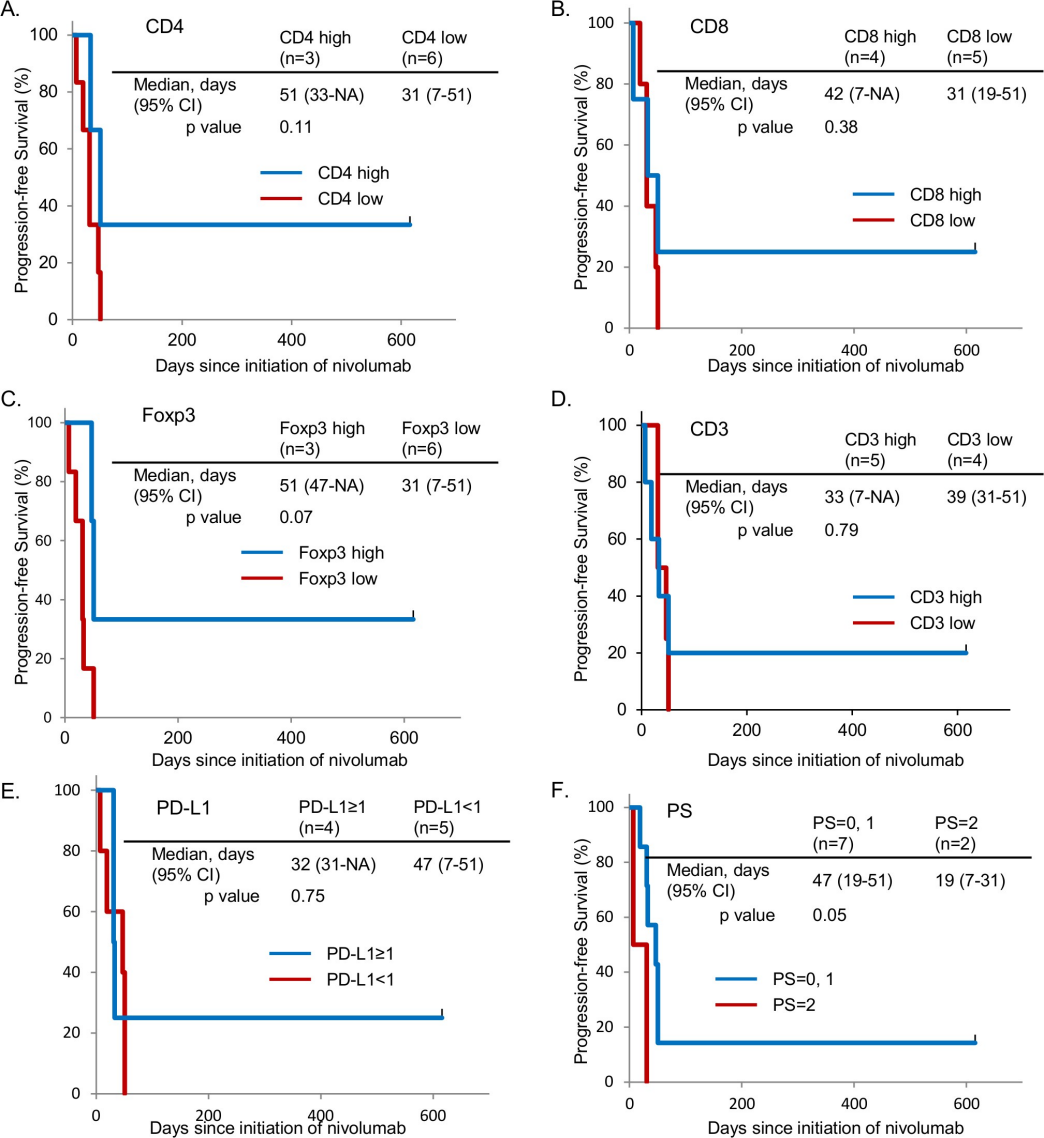
Progression-free survival curves according to potential predictive biomarkers for nivolumab. Kaplan-Meier curves are shown for the patients with CD4^+^ high vs. low (A), CD8^+^ high vs. low (B), Foxp3^+^ high vs. low (C), CD3^+^ high vs. low (D), PD-L1 positive vs. negative (E) and PS 0, 1 vs. 2 (F).

We were not able to evaluate the correlation of these biomarkers and OS because only 3 out of 9 patients had died of lung cancers. The Kaplan-Meier curves for PFS for CD4^+^ high vs. low, CD8^+^ high vs. low, Foxp3^+^ high vs. low, CD3^+^ high vs. low, PD-L1 positive vs. negative and PS 0 and 1 vs. PS 2 are shown in Fig 3. Consistent with the results of multivariate analyses, PFS tended to be longer among patients with a high number of CD4^+^ TILs and a high number of Foxp3^+^ TILs. Different from the results of multivariate analyses, patients with PS 0 and 1 had a longer survival time compared to that of patients with PS 2. Table 4 shows individual data of patients in this study.

**Table 4:** Individual data of all study patients (n = 9).

Case	Age (years)	Sex	Smoking status	Types of EGFR mutation	Prevoius treatmet lines before nivolumab	EGFR-TKIs	PD-L1 expression	No. of CD4^+^ T cells (/mm^2^)	N0. of CD8^+^ T cells (/mm^2^)	No. of Foxp3^+^ T cells (/mm^2^)	PS	NLR	Response to nivolumab	PFS (days)	OS (days)
1	37	F	Never	19del. +T790M	5	G→E→A	0%	2882	156	44	1	2.4	PD	19	469
2	40	M	Never	19del.	2	Gefitinib	100%	2248	978	8	1	1.96	PD	33	370
3	62	F	Never	19del.	3	Gefitinib	0%	3452	434	22	1	1.96	PD	51	480
4	66	F	Never	20 insertion	4	None	30–40%	5567	1405	874	1	1.51	PR	616	616
5	62	F	Never	19del.	2	Afatinib	0%	951	1117	508	1	2.13	PD	51	355
6	72	M	Former	19del.	2	Afatinib	0%	1548	236	227	1	2.17	PD	47	299
7	62	F	Never	L858R +T790M	3	Afatinib	5–9%	1502	373	11	0	9.875	NE	31	361
8	40	M	Former	S768I	2	None	0%	1942	1186	22	0	4.94	PD	7	44
9	62	F	Never	19del.	1	Afatinib	1–4%	1407	309	54	1	3.44	PD	31	230

F, female; M, male; G, gefitinib; E, erlotinib; A, afatinib; PS, performancde status; NLR, netrophil to lymphocyte ratio; PFS, progression-free survival; OS, overall survival

## Discussion

Previous clinical trials have suggested that PD-1/PD-L1 blockade therapies are less effective for patients with *EGFR* mutations than for patients with wild-type *EGFR* [5, 21, 29, 30]. Meta-analysis of randomized trials comparing anti-PD-1/PD-L1 inhibitors with docetaxel revealed that patients with *EGFR* mutations did not benefit from PD-1/PD-L1 blockade therapies in terms of OS, and PFS was even worse [31]. Thus, predictive biomarkers are required to improve the outcomes of PD-1/PD-L1 blockade therapies in *EGFR*-mutated NSCLC patients. The expression of PD-L1 in the tumor microenvironment is the most commonly investigated biomarker for anti-PD-1/PD-L1 treatments. The association of clinical benefits with PD-L1 expression has been demonstrated in PD-1/PD-L1 blockade therapies [32]. However, it is controversial whether PD-L1 expression is also useful to predict the antitumor effects of PD-1/PD-L1 inhibitors in *EGFR*-mutated NSCLCs. In a prospective phase II trial, pembrolizumab, which is an anti-PD-1 antibody, failed to show clinical benefits in PD-L1 positive *EGFR*-mutated NSCLC patients, even in those with a high expression of PD-L1 [33]. In the current study, 4 out of 9 patients were PD-L1 positive, and a correlation between PD-L1 expression and clinical efficacy was not observed (Table 3 and Fig 3). Further, there was no statistical difference of PFS with nivolumab between patients with PD-L1 tumor proportion score of 50% or greater and patients with PD-L1 tumor proportion score of less than 50% (data not shown). Tumor cells express PD-L1 in response to inflammatory cytokines, such as IFN-γ, to escape from attack by effector T cells. Recent studies reported that PD-L1 expression is induced by signaling through EGFR [34, 35]. This finding may be the reason why PD-L1 expression is not a reliable predictive biomarker in patients with *EGFR* mutations.

Accumulating evidence suggests that an inflamed tumor microenvironment may predict clinical benefits for PD-1/PD-L1 blockade therapies. Considering the mechanisms of PD-1/PD-L1 blockade therapies, the existence of effector T cells that are suppressed through the PD-1/PD-L1 axis could be a good predictive biomarker for PD-1/PD-L1 inhibitors. Indeed, several studies reported that the density of CD8^+^ T cell infiltration in tumor tissues was associated with the effectiveness of PD-1/PD-L1 targeted therapies [9, 11]. The high expression of gene signatures, which were associated with effector T cells and IFN-γ, were also correlated with the effectiveness of anti-PD-L1 treatment [36]. Wu et al. further demonstrated that the high frequency of PD-L1^+^CD4^+^CD25^+^ Tregs predicted better outcomes in patients treated with PD-1/PD-L1 blockade therapies [11]. Because the expression of PD-1 on Tregs has a critical role in maintaining their suppressive function, anti-PD-1 treatment may improve immune responses in the tumor microenvironment by inhibiting the function of Tregs [37, 38]. In the current study, the high density of CD4^+^ T cells and Foxp3^+^ Treg cells, but not CD8^+^ T cells, in the tumor microenvironment was positively correlated with better PFS (Table 3 and Fig 3). As discussed above, *EGFR*-mutated NSCLCs might express PD-L1 by signaling through EGFR and/or in response to inflammatory cytokines from effector T cells. The population of immune cells infiltrating tumor tissues may be good predictive determinants of PD-1/PD-L1 blockade therapies.

After failure of EGFR-TKI treatment, the mechanisms of immune escape in NSCLCs with *EGFR* mutations might be different from those in NSCLCs with *EGFR* mutations prior EGFR-TKI treatment. Epithelial-mesenchymal transition and cMET amplification, which were reported to be acquired resistance suppressed CD8^+^ T cells [39, 40]. Haratani et al also demonstrated that compared to T790M-positive NSCLCs, T790M-negative NSCLCs had a higher level of PD-L1 expression and tended to be benefit from anti-PD-1 treatment [41]. The information about acquired resistance to EGFR-TKIs may be helpful to guide the administration of PD-1/PD-L1 inhibitors for *EGFR*-mutated NSCLCs. In our study, only one patient who had an exon 20 insertion and was previously untreated with EGFR-TKIs showed a durable response to nivolumab. Adequate timing of PD-1/PD-L1 blockade therapies for *EGFR*-mutated NSCLCs and the association between types of EGFR mutation, resistance mechanisms to EGFR-TKIs and response to PD-1/PD-L1 blockade therapies remain to be elucidated.

The limitations of the present study include its retrospective design and small sample size. Because only one patient responded to nivolumab in this study, the results of univariate and multivariate analyses could be strongly affected by the immune status of this patient. Further studies are necessary to confirm the correlation between clinical efficacy of PD-1/PD-L1 antibodies and the density of CD4^+^ T cells and Foxp3^+^ Treg cells in the tumor microenvironment. In addition, we only analyzed *EGFR*-mutated NSCLC patients. Now we are evaluating surface markers of TILs in *EGFR* wild-type NSCLC patients to clarify whether the density of CD4^+^ T cells and Foxp3^+^ T cells is correlated with the efficacy of anti-PD-1/PD-L1 treatment.

In conclusion, our findings demonstrated that patients with *EGFR* mutations poorly responded to nivolumab treatment regardless of PD-L1 expression on tumor cells. The immune status of tumor microenvironment may predict antitumor effects of nivolumab in patients with *EGFR* mutations. Further studies are warranted to identify predictive biomarkers for anti-PD-1/PD-L1 antibodies in *EGFR*-mutated NSCLC patients.

## Supporting information

S1 File Individual data of all study patients. (XLSX)
